# Associations of prenatal blood pressure trajectory and variability with child neurodevelopment at 2 years old

**DOI:** 10.1186/s12916-024-03439-3

**Published:** 2024-05-30

**Authors:** Luli Xu, Jiayi Cheng, Xiaohan Dong, Menglan Guo, Kai Chen, Xiaoxuan Fan, Xiaofeng Mu, Yuji Wang, Zhiguo Xia, Jun Li, Youjie Wang, Chao Xiong, Aifen Zhou

**Affiliations:** 1 Wuhan Children’s Hospital (Wuhan Maternal and Child Healthcare Hospital), Tongji Medical College, Huazhong University of Science and Technology, Wuhan, Hubei China; 2 Department of Maternal and Child Health, School of Public Health, Tongji Medical College, Huazhong University of Science and Technology, Wuhan, 430030 China

**Keywords:** Blood pressure multi-trajectory, Blood pressure variability, Child neurodevelopment, Group-based multi-trajectory model, Birth cohort study

## Abstract

**Background:**

The patterns of blood pressure (BP) change throughout the pregnancy were related to adverse birth outcomes. However, little is known about the long-term effect of BP change patterns on child neurodevelopment. This study aimed to explore the relationship between the BP trajectory and BP variability during pregnancy and early childhood neurodevelopment.

**Method:**

A total of 2797 mother-newborn pairs were derived from the Wuhan Healthy Baby Cohort Study. BP was measured during each antenatal visit, and Mental and Psychomotor Development Indexes (MDI and PDI) were assessed using the Bayley Scales of Infant Development (BSID) when the children were 2 years old. Delayed neurodevelopment was defined as scores of PDI or MDI less than − 1SD relative to the mean score of the study population. A group-based multi-trajectory model was adopted to identify multi-trajectories of systolic blood pressure (SBP) and diastolic blood pressure (DBP). Visit-to-visit BP variability was assessed by the coefficient of variation (CV), standard deviation (SD), and average real variability (ARV). Generalized linear models and multivariate logistic regressions were used to assess the associations of BP trajectories and variability with BSID scores and delayed neurodevelopment, respectively.

**Results:**

Five distinct trajectories for SBP and DBP were identified, namely, “Low-increasing,” “Low-stable,” “Moderate-decreasing,” “Moderate-increasing,” and “High-stable” groups. Compared with the “Low-stable” group, the children whose mothers’ BP fell into the other four groups had lower PDI scores, and mothers in the “Low-increasing,” “Moderate-increasing,” and “Moderate-decreasing” groups had 43% (OR: 1.43, 95% CI: 1.01, 2.03), 48% (OR: 1.48, 95% CI: 1.05, 2.08) and 45% (OR:1.45, 95% CI: 1.03, 2.04) higher risk of having offspring with delayed psychomotor neurodevelopment, respectively. High DBP variability was associated with lower BSID scores, and delayed psychomotor neurodevelopment (OR = 1.46, 95% CI: 1.10, 1.92 for DBP-SD; OR = 1.53, 95% CI: 1.16, 2.02 for DBP-CV).

**Conclusion:**

Our findings suggest that BP change patterns assessed by multi-trajectory and visit-to-visit variability were associated with lower BSID scores and delayed neurodevelopment. Health professionals should be aware of the influence of BP level and its oscillations during pregnancy on the risk of delayed neurodevelopment.

**Supplementary Information:**

The online version contains supplementary material available at 10.1186/s12916-024-03439-3.

## Background

Hypertensive disorders of pregnancy (HDP), which include chronic hypertension and the development of concurrent hypertension in pregnancy, are common pregnancy complications [1]. Growing studies have shown that HDP can create adverse condition in utero, potentially impacting fetal development and leading to long-term consequences for vascular, cognitive, and neurodevelopmental outcomes in offspring [2–4]. Typically HDP is diagnosed clinically based on the highest blood pressure (BP) measurements during the regular antenatal check-ups, however, assessing BP status and dynamic variation based on a single time point has limitations due to the influence of behavioral, emotional, and environmental factors. Previous research has examined the patterns of BP change during pregnancy. For example, a study conducted in Suzhou, China, analyzed the BP trajectories in 28,679 pregnant women and found that the trajectories of both SBP and DBP followed the same trend throughout pregnancy, labeled as low-stable, moderate-increasing, moderate-decreasing, and high-stable [5]. In another study from Japan, 755 pregnant women used home monitoring to track their BP, leading to the identification of six trajectory groups for both SBP and DBP during pregnancy: low-J-curve, low-steep J-curve, moderate J-curve, moderate–reverse J-curve, little high J-curve, and high J-curve [6]. Additionally, Malaba et al. identified joint trajectory groups based on a combination of SBP and DBP trajectory in pregnant women from South Africa, resulting in groups labeled as consistent low normal, consistent normal, consistent high normal, and increasing abnormal [7]. These findings underscore the importance of adopting a longitudinal approach to BP measurement in research settings, akin to clinical practice. Such an approach involves assessing BP patterns throughout pregnancy rather than relying on single-point measures.

Previous studies have shown that abnormal BP status during pregnancy can affect placental perfusion, potentially reducing oxygen and nutrient supply to the fetus and resulting in alterations in fetal brain development and subsequent behavioral and cognitive deficits [2, 8]. Previous studies suggested that HDP were associated with neurodevelopment delay at early childhood, and even had an impact on neurodevelopmental disorders in offspring, including autism spectrum disorders, attention-deficit/hyperactivity disorder, and intellectual disability [9, 10]. Furthermore, even elevated BP within the normal range may affect child neurodevelopment. Liu et al. reported that maternal third-trimester BP levels (both systolic BP and diastolic BP) were associated with a decrease in Bayley Scale of Infant Development scores among 1008 mother–child pairs in China [11]. However, most studies have only considered BP at a single time point and have not explored the patterns of BP change throughout pregnancy. Clinical evidence supports the notion that BP changes in trajectory [6, 7, 12] and variability [13–15] are related to adverse birth outcomes, such as preterm delivery, low birth weight, and fetal distress, but the association of BP trajectory and variability with early childhood neurodevelopment remains largely unknown.

Given the correlations between systolic blood pressure (SBP) and diastolic blood pressure (DBP), a single indicator trajectory of SBP or DBP is insufficient to capture the potential synergistic effect of SBP and DBP. In this study, a multivariate group-based trajectory model was used to identify multi-trajectories of SBP and DBP, allowing for the identification of subgroups with shared profiles of both SBP and DBP. Based on a birth cohort, we aimed to determine multi-trajectories and visit-to-visit variability of SBP and DBP throughout antenatal examinations and explore the association between BP change patterns and early childhood neurodevelopment.

## Methods

### Study population

**Fig. 1:**
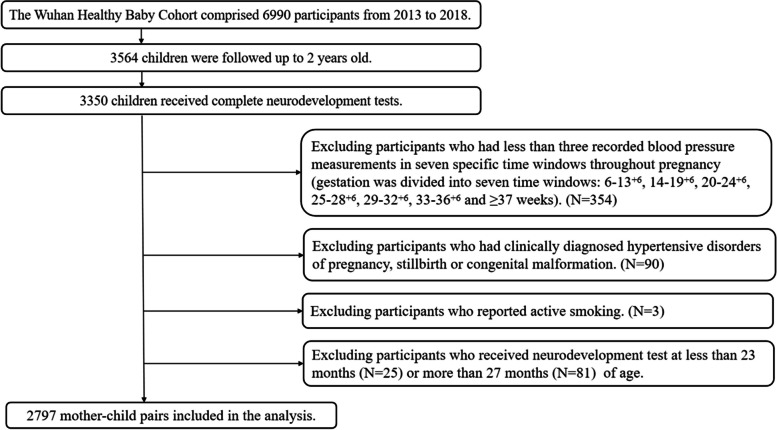
Flow chart of the study population

The participants in this study were taken from the Wuhan Healthy Baby Cohort (WHBC) Study, which was a birth cohort established in Wuhan, China. Briefly, from 2013 to 2018, we recruited participants who: (1) were less than 16 weeks pregnant at recruitment; (2) had a singleton pregnancy; (3) were scheduled to receive prenatal care and delivery at Wuhan Children's Hospital (Wuhan Maternal and Child Health Hospital). Trained nurses conducted face-to-face interviews with each participant using a structured questionnaire to collect information on demographics, lifestyle, health conditions, and obstetric history. Maternal BP was measured at each antenatal visit. Neurodevelopment tests were performed at the study hospitals by certified psychologists when the children were 2 years old. The Wuhan Healthy Baby Cohort comprised 6990 participants from 2013 to 2018. Of these, 3564 children were followed up to 2 years old, and 3350 children received complete neurodevelopment tests. In the current study, we excluded participants who (1) had less than three recorded blood pressure measurements in seven specific time windows throughout pregnancy; (2) had clinically diagnosed hypertensive disorders of pregnancy, stillbirth, or congenital malformation; (3) reported active smoking during pregnancy; and (4) had not received neurodevelopment test at age of 2 years (range 23–26 months). Finally, there were 2797 mother–child pairs included in the analysis (Fig. 1).

The research protocol was approved by the Medical Ethics Committee of Wuhan Children’s Hospital (Wuhan Maternal and Child Healthcare Hospital), Tongji Medical College, Huazhong University of Science and Technology (Approve Number: 2010009). All participants were informed and signed an informed consent form.

### Blood pressure measurements during pregnancy

During each antenatal visit, BP was measured on the right upper arm by a certified nurse using a calibrated mercury sphygmomanometer after the participants rested in the sitting position for 5 min. Blood pressure was measured twice at an interval over 1 min, and the mean value of two blood pressure readings was documented.

### Child neurodevelopment

At 24 months of age (mean [standard deviation (SD)] age at assessment, 23.85 [0.81] months), children’s neurodevelopment was assessed by the adapted Chinese revision of the Bayley Scale of Infant Development (BSID-I), which has been validated with good consistency and reliability [16]. It generates two indices expressed as the mental development index (MDI) and the psychomotor development index (PDI). The MDI is used to assess a total of 163 items related to cognitive abilities, and the PDI assesses a total of 81 items related to motor abilities. Raw scores of BSID assessments were calculated and converted to index scores of MDI and PDI with an average of 100 and a standard deviation (SD) of 15. All BSID assessments were standardized according to the age of the participants. Scores less than 1 SD below the mean of either the PDI or MDI of the children in this study were defined as delayed psychomotor or mental neurodevelopment, respectively [17].

### Covariate assessment

Information on demographics and lifestyle was collected via face-to-face interviews using questionnaires, including maternal age, educational level (junior high school or below, high school, college or above), and passive smoking status during pregnancy (yes/no). Maternal health history and obstetric history were obtained from medical records. Pre-pregnancy body mass index (BMI) was calculated by dividing pre-pregnancy weight in kg (self-reported) by the height in squared meters (measured during the prenatal visit) (weight/height^2^). Information about newborns on the gestational week and sex was ascertained from birth records. Small for gestational age (SGA) was defined as the newborns whose birth weight was below the 10th percentile of average weight for the same gestational age [18].

### Statistical analysis

The missing rates for pre-pregnancy BMI and passive smoking during pregnancy were 0.04% and 0.64%, respectively. Other variables such as maternal age, educational level, gestational week, infant sex, birth weight, and gestational diabetes mellitus (GDM) were complete. To address missing values, we employed multiple imputation using an alternative modeling strategy of Markov chain Monte Carlo (MCMC). The results across 5 imputed datasets were combined by averaging, ensuring robustness and reliability in our analysis. Continuous and categorical variables were expressed as the mean with SD and frequencies with percentages, respectively. Given the repeated BP measurements taken during different stages of pregnancy, 7 gestational age (GA) time point windows were established based on the Chinese Guideline for Preconception and Prenatal Care (2018) [19]. These time points were 6–13^+6^, 14–19^+6^, 20–24^+6^, 25–28^+6^, 29–32^+6^, 33–36^+6^ and ≥ 37 weeks of gestation. For each GA time point window, the BP measurement closest to the highest frequency GA time point was selected, taking into account the distribution of GA at the actual prenatal visit. Any remaining BP measurements in this window were discarded.

To identify subgroups of participants with similar trajectories in SBP and DBP, group-based multi-trajectory modeling (GBMTM) was applied. This approach allows for capturing the correlation and the potential synergistic effect of SBP and DBP. The optimal GBMTM model was determined based on the following criteria: (1) improvement in the Bayesian information criterion; (2) ensuring that each trajectory group contained more than > 5% of the participants; (3) high average posterior probabilities of group membership (> 0.7) [20]. Subject-matter knowledge was also used to make decisions about the optimal number of groups.

Three metrics were used to define visit-to-visit BP variability: the coefficient of variation (CV), SD, and average real variability (ARV) of both SBP and DBP, as calculated according to the following equations: $\text{CV}=\frac{\text{SD}}{\textrm{mean}}*100\% , \text{SD}=\sqrt{\frac{\sum_{i=1}^{n}{({x}_{i}-\overline{x })}^{2}}{n-1}}, \text{ARV}=\frac{1}{n-1}{\sum }_{k=1}^{n-1}\left|{C}_{k+1}-{C}_{k}\right|$, where *C* is the value of BP, *k* ranges from 1 to *n* − 1, and *n* is the number of measurements for BP. Each metric was categorized into tertile (low, intermediate, and high variability). Generalized linear models and multivariate logistic regressions were used to assess the relationship between BP trajectories/variability and BSID scores as well as the occurrence of delayed neurodevelopment, respectively. Covariates were ascertained according to prior published literature and the change in estimate method (if the estimate changed by > 10%, the variable was entered in the model), including maternal age, pre-pregnancy BMI, educational level, passive smoking status, SGA, infant sex, the number of BP measurements, and GDM [5, 14, 21]. Additionally mean BP levels were also adjusted for in the models when assessing the association of BP variability and neurodevelopment. Considering the effect of preterm birth, the model that only adjusts for confounders (maternal age, pre-pregnancy BMI, educational level, passive smoking status, infant sex, the number of BP measurements, and GDM) and an additional model that adjusts for gestational age were provided in the Supplementary materials (Additional file 1: Table S1-S4).

Analyses were performed using SAS 9.4 (SAS Institute Inc., Cary, NC), with GBMTM conducted using PROC TRAJ macros. The significance level was set at 0.05.

## Result

### Basic characteristics

Baseline characteristics of 2797 mother-newborn pairs are displayed in Table 1. The mean of age at recruitment and pre-pregnancy BMI for mothers was 28.91 ± 3.57 years and 20.87 ± 2.79 kg/m^2^, respectively. More than half of the participants (50.55%) had an education level of college or above. A small proportion of mothers (4.76%) had been exposed to secondhand smoke during pregnancy. Among the children, 1445 (51.66%) were male. The average gestational week was 39.37 ± 1.09 weeks. Most participants had six (33.04%) or seven (30.68%) BP measurements within the designated time windows. Summary statistics for BP measurements at each indicated gestational window are shown in Table 2. The BP generally tended to increase throughout pregnancy.

**Table 1:** Descriptive characteristics of participants

Characteristics	All individuals (*n* = 2797)
**Mothers**	
Maternal age (years)	28.91 ± 3.57
Pre-pregnancy BMI (kg/m^2^)	20.87 ± 2.79
Maternal educational level, *n* (%)	
Junior high school or below	549 (19.63)
High school	834 (29.82)
College or above	1414 (50.55)
Passive smoking during pregnancy, *n* (%)	
Yes	133 (4.76)
No	2664 (95.24)
Parity, *n* (%)	
Primiparous	2276 (81.37)
Multiparous	521 (18.63)
Gestational diabetes mellitus, *n* (%)	
Yes	257 (9.19)
No	2540 (90.81)
The number of BP measurements	
Three	121 (4.33)
Four	278 (9.94)
Five	616 (22.02)
Six	924 (33.04)
Seven	858 (30.68)
**Newborns**	
Infant sex, *n* (%)	
Male	1445 (51.66)
Female	1352 (48.34)
Birth weight (kg)	3.34 ± 0.41
Birth length (cm)	50.37 ± 1.53
Gestational week (weeks)	39.37 ± 1.09
**BSID assessment**	
Age at assessment, month	23.85 ± 0.81
PDI score	107.83 ± 18.25
MDI score	107.93 ± 22.11

**Table 2:** Summary statistics for BP measurements at each indicated gestational window

Time windows	*N*	SBP (mmHg)	DBP (mmHg)
6–13^+6^ week	2658	110.53 ± 10.74	68.88 ± 9.03
14–19^+6^ week	2016	110.68 ± 10.39	69.24 ± 8.93
20–24^+6^ week	2171	110.87 ± 10.48	68.83 ± 8.81
25–28^+6^ week	2002	110.93 ± 10.50	68.92 ± 8.89
29–32^+6^ week	2384	111.23 ± 10.31	69.25 ± 8.86
33–36^+6^ week	2512	112.94 ± 10.54	70.48 ± 9.21
≥ 37 week	2362	113.63 ± 10.52	71.18 ± 9.20

### Multi-trajectories of blood pressure during pregnancy

**Fig. 2:**
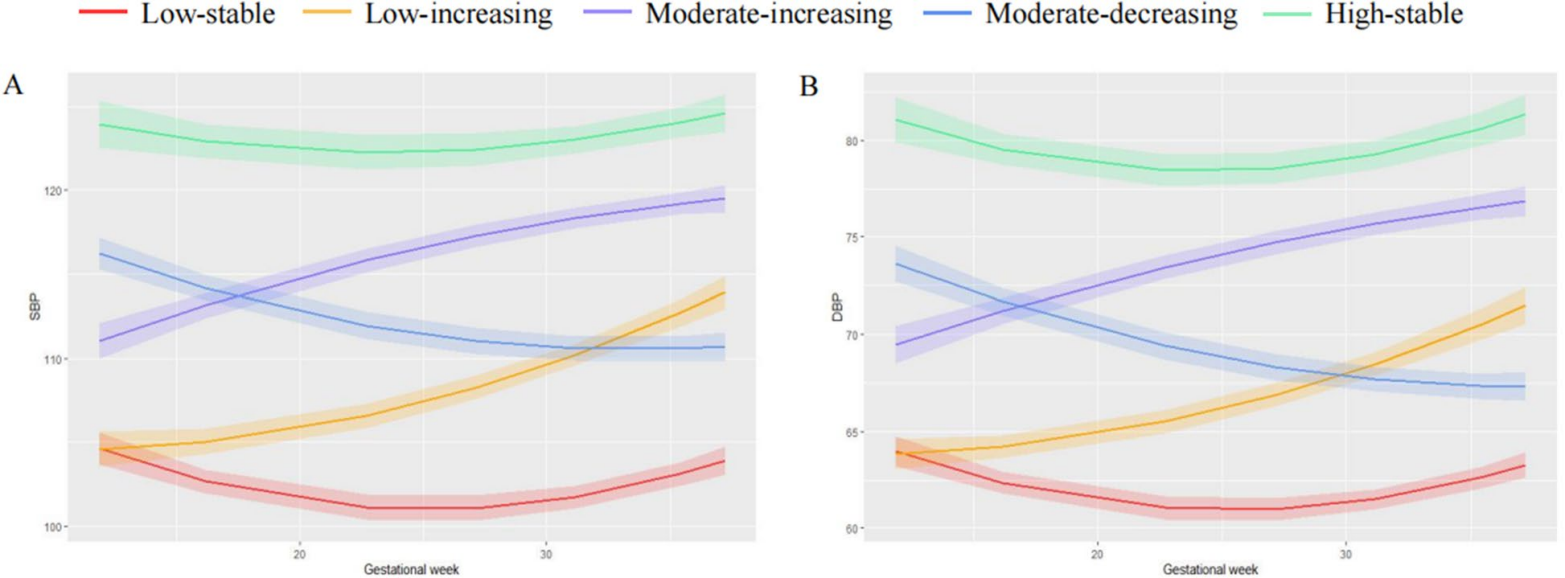
Multi-trajectories of systolic blood pressure (**A**) and diastolic blood pressure (**B**). Figure shows five multi-trajectories of SBP and DBP which were identified from group-based multi-trajectory modeling. The solid line and shaded portion represented point estimation and 95% CI estimation, respectively. *SBP* systolic blood pressure, *DBP* diastolic blood pressure

Using a multivariate trajectory modeling approach, a 5-group solution was determined as optimal to describe trajectories of BP (Table 3). Figure 2A and B shows the five multi-trajectory curves of SBP and DBP, respectively. Pregnant women in the “Low-stable” group (*n* = 494, 17.66%) had the lowest BP levels and remained relatively stable, showing a slight U-shaped curve. The “Low-increasing” group (*n* = 659, 23.56%) had the lowest BP in early pregnancy but showed a sharp increase by a J-shaped curve until late pregnancy. Two groups, “Moderate-increasing” (*n* = 746, 26.67%) and “Moderate-decreasing” (*n* = 685, 24.49%), had average BP levels in early pregnancy with completely opposite trends throughout pregnancy. The “Moderate-increasing” groups showed a gradual slowdown in the rate of increase, while the “Moderate-decreasing” group exhibited a reverse J-shaped curve. Additionally, a “High-stable” group (*n* = 213, 7.62%) was identified with the highest BP throughout pregnancy and a slight change of U-shaped curve, maintaining a relatively stable overall trend.

**Table 3:** Descriptions of multivariate trajectory groups identified by group-based multi-trajectory models

Group	Name	Description
1	Low-stable	Pregnant women in this group had the lowest blood pressure levels and remained relatively stable, showing a slight U-shaped curve
2	Low-increasing	The blood pressure in this group was low in early pregnancy, with a sharp increase in SBP and DBP, showing a J-shaped curve until late pregnancy
3	Moderate-increasing	The blood pressure in this group was average in early pregnancy and increased throughout pregnancy with a gradual slowdown in the rate of increase
4	Moderate-decreasing	The blood pressure in this group was average in early pregnancy and decreased throughout pregnancy, showing a reverse J-shaped curve
5	High-stable	The blood pressure in this group was the highest throughout pregnancy, with a slight change of U-shaped curve, maintaining a relatively stable trend overall

### Association of trajectories of blood pressure and child neurodevelopment

The associations of BP trajectory with BSID score and delayed neurodevelopment are presented in Table 4. Compared with the “Low-stable” group, the children whose mothers belong to the other four groups had lower PDI scores, the *β* (95% CI) values were − 2.94 (− 5.09, − 0.79) for the “Low-increasing” group, − 3.21 (− 5.31, − 1.10) for the “Moderate-increasing” group, − 3.12 (− 5.21, − 1.03) for the “Moderate-decreasing” group, and − 3.39 (− 6.42, − 0.35) for the “High-stable” group, after adjusting for maternal age, pre-pregnancy BMI, educational level, passive smoking status, SGA, infant sex, and GDM. No association was observed between BP trajectories and MDI scores.

**Table 4:** Associations between multivariate trajectory groups for blood pressure and child neurodevelopment

	BSID score		Delayed neurodevelopment	
	PDI	MDI	PDI	MDI
Low-stable	ref	ref	ref	ref
Low-increasing	** − 2.94 (− 5.09, − 0.79)**	− 0.80 (− 3.35, 1.75)	**1.43 (1.01, 2.03)**	1.12 (0.80, 1.57)
Moderate-increasing	** − 3.21 (− 5.31, − 1.10)**	− 0.47 (− 2.98, 2.03)	**1.48 (1.05, 2.08)**	1.11 (0.79, 1.55)
Moderate-decreasing	** − 3.12 (− 5.21, − 1.03)**	− 2.31 (− 4.79, 0.17)	**1.45 (1.03, 2.04)**	1.22 (0.88, 1.69)
High-stable	** − 3.39 (− 6.42, − 0.35)**	− 2.53 (− 6.13, 1.06)	1.41 (0.87, 2.30)	1.18 (0.74, 1.89)

The models were adjusted for maternal age, educational level, pre-pregnancy BMI, passive smoking status, small for gestational age, infant sex, the number of BP measurements, and gestational diabetes mellitus

*BSID* Bayley Scale of Infant Development, *PDI* psychomotor development index, *MDI* mental development index

Among the total 2797 children, 421 (15.05%) were classified as having delayed psychomotor neurodevelopment, and 437 (15.62%) were classified as having delayed mental neurodevelopment. Using the “Low-stable” group as reference, mothers in the “Low-increasing,” the “Moderate-increasing,” and the “Moderate-decreasing” groups had 43% (OR: 1.43, 95% CI: 1.01, 2.03), 48% (OR: 1.48, 95% CI: 1.05, 2.08), and 45% (OR:1.45, 95% CI: 1.03, 2.04) higher risk of giving birth to children with delayed psychomotor neurodevelopment, respectively, after adjusting for potential covariates. No association was observed between BP trajectories and delayed mental neurodevelopment.

### Association between blood pressure variability and child neurodevelopment

We further assessed the association of the BP variability with PDI and MDI scores and delayed neurodevelopment (Table 5). After adjusting for maternal age, pre-pregnancy BMI, educational level, passive smoking, SGA, infant sex, the number of BP measurements, GDM, and mean DBP, the PDI score was negatively associated with DBP variability assessed by DBP-SD (*β*: − 2.20, 95% CI: − 4.05, − 0.35) and DBP-CV (*β*: − 2.39, 95% CI: − 4.25, − 0.53). Mothers with high DBP-SD tended to have children with lower MDI scores (*β*: − 2.31, 95% CI: − 4.48, − 0.15). No statistically significant association was observed between SBP variability with PDI or MDI scores. The children whose mothers had high DBP variability exhibited a higher risk of delayed psychomotor neurodevelopment (OR: 1.46, 95% CI: 1.10, 1.92 for DBP-SD; OR: 1.53, 95% CI: 1.16, 2.02 for DBP-CV). No association was observed between BP variability and delayed mental neurodevelopment.

**Table 5:** Associations between blood pressure variability and child neurodevelopment

	BSID score		Delayed neurodevelopment	
	PDI	MDI	PDI	MDI
SBP-SD (mmHg)				
Low (< 5.94)	ref	ref	ref	ref
Intermediate (5.94–8.29)	0.07 (− 1.79, 1.94)	0.21 (− 1.98, 2.40)	0.95 (0.72, 1.26)	1.01 (0.76, 1.35)
High (> 8.29)	0.12 (− 1.72, 1.95)	− 0.01 (− 2.16, 2.13)	0.88 (0.67, 1.16)	0.99 (0.75, 1.32)
SBP-CV (%)				
Low (< 5)	ref	ref	ref	ref
Intermediate (5–7)	0.37 (− 1.48, 2.23)	− 0.57 (− 2.74, 1.61)	0.92 (0.70, 1.21)	1.09 (0.82, 1.44)
High (> 7)	0.77 (− 1.06, 2.60)	0.08 (− 2.07, 2.23)	0.85 (0.65, 1.12)	0.94 (0.71, 1.25)
SBP-ARV (mmHg)				
Low (< 7.0)	ref	ref	ref	ref
Intermediate (7.0–10.4)	0.97 (− 0.88, 2.83)	− 0.72 (− 2.89, 1.46)	0.98 (0.74, 1.30)	1.13 (0.85, 1.50)
High (> 10.4)	− 1.43 (− 3.27, 0.40)	− 0.99 (− 3.14, 1.17)	1.06 (0.80, 1.39)	1.09 (0.82, 1.45)
DBP-SD (mmHg)				
Low (< 5.17)	ref	ref	ref	ref
Intermediate (5.17–7.24)	− 0.70 (− 2.59, 1.19)	− 1.36 (− 3.57, 0.86)	1.13 (0.84, 1.52)	1.06 (0.79, 1.41)
High (> 7.24)	** − 2.20 (− 4.05, − 0.35)**	** − 2.31 (− 4.48, − 0.15)**	**1.46 (1.10, 1.92)**	1.17 (0.88, 1.55)
DBP-CV (%)				
Low (< 7)	ref	ref	ref	ref
Intermediate (7–11)	− 0.91 (− 2.80, 0.98)	− 1.51 (− 3.72, 0.71)	1.06 (0.79, 1.43)	1.04 (0.77, 1.39)
High (> 11)	** − 2.39 (− 4.25, − 0.53)**	− 2.17 (− 4.35, 0.01)	**1.53 (1.16, 2.02)**	1.15 (0.87, 1.53)
DBP-ARV (mmHg)				
Low (< 6.0)	ref	ref	ref	ref
Intermediate (6.0–9.0)	0.95 (− 0.93, 2.83)	− 0.90 (− 3.10, 1.30)	0.82 (0.61, 1.08)	1.10 (0.83, 1.47)
High (> 9.0)	− 0.11 (− 1.98, 1.76)	− 1.61 (− 3.81, 0.58)	1.02 (0.78, 1.34)	1.07 (0.81, 1.43)

Each parameter was categorized into tertiles, with tertile 1, tertile 2, and tertile 3 considered as low, intermediate, and high BP variability, respectively. The models were adjusted for maternal age, educational level, pre-pregnancy BMI, passive smoking status, small for gestational age, infant sex, the number of BP measurements, gestational diabetes mellitus, and mean SBP or DBP

*BSID* Bayley Scale of Infant Development, *PDI* psychomotor development index, *MDI* mental development index, *SBP *systolic blood pressure, *DBP* diastolic blood pressure, *SD* standard deviation, *CV* coefficient of variation, *ARV* average real variability

## Discussion

The current study demonstrated that long-term patterns of BP changes, determined using BP trajectories and visit-to-visit variability, were associated with early childhood neurodevelopment in a prospective birth cohort. Several BP trajectories during pregnancy were significantly associated with lower PDI scores and a higher risk of delayed psychomotor neurodevelopment. In addition, high DBP variability was associated with lower both PDI and MDI scores and delayed psychomotor neurodevelopment.

Previous studies have explored the separate SBP and DBP trajectories during pregnancy, however, patterns of co-evolution of both SBP and DBP have been rarely investigated. Malaba et al. applied the group-based trajectory modeling to identify categories of pregnant women characterized by different joint trajectories of SBP and DBP during pregnancy. Specifically, women were assigned to “joint groups” characterized by different combinations of SBP and DBP trajectories using a probability matrix of joint category membership and consideration of the clinical significance of observed differences [7]. Furthermore, the GBMTM, a multi-trajectory model, was used to identify synergistic changes in systolic and diastolic hypertension and fitted novel, reasonable, and clinically defined trajectories [22]. This study firstly applied the GBMTM to investigate the pattern of synergistic changes in SBP and DBP during pregnancy, producing interpretable results consistent with clinical reality. The trends of SBP and DBP in each group remained generally consistent throughout pregnancy and were identified as five groups, namely “Low-stable,” “Low-increasing,” “Moderate-increasing,” “Moderate-decreasing,” and “High-stable” group. Previous studies have shown that blood pressure decreases from pre-pregnancy levels to a nadir during early pregnancy and then rises before delivery, following a U-shape curve [23, 24]. In the present study, pregnant women in the “Low-stable” trajectory group maintained their blood pressure at a low level throughout pregnancy and showed a U-shaped curve, which was used as a reference group in subsequent analyses. The “High-stable” group had the highest BP levels throughout gestation with an inclined U-shaped change. Pregnant women in the other three trajectory groups experienced considerable changes during pregnancy. Our findings are consistent with previous researches conducted in Suzhou, China [19], and Japan [5], which identified similar patterns of BP trajectory in pregnant women. The BP patterns during pregnancy are influenced by a variety of factors, including environmental, behavioral, and biological variables. It’s important to note that our study design had limitations regarding the collection of all potential risk factors impacting BP levels. Seasonal variations could have influenced the observed differences among BP trajectories. Cold-induced mechanisms likely contributed to the overall effect, with winter associated with higher BP levels. Additionally, factors such as lower vitamin D levels, reduced activity, and weight gain may have contributed to these trends [23].

Notably, even though BP changes in different trajectories fall within the physiological range of normal BP, pregnant women with a low-increasing, moderate-increasing, and moderate-decreasing pattern had an increased risk of delayed psychomotor neurodevelopment in children at 2 years. Previous researches have also found an association between several BP long-term patterns and lower birth weight and other adverse birth outcomes even when the BP values throughout pregnancy were below the diagnostic threshold for hypertension [5, 6]. These results suggest that BP fluctuations within the normal range during pregnancy were associated with neurodevelopment of offspring. Zhu et al. reported a significant dose–response relationship between BP within the normal range and the risk of adverse birth outcome during pregnancy [24]. Liu et al. indicated that increased maternal normal range BP may affect child neurodevelopment [11]. A large population-based study concluded that among normotensive women, higher SBP and DBP during the second and third trimesters were positively associated with offspring overweight/obesity risk [25]. Our findings, combined with previous studies, suggest that pregnant women require appropriate blood pressure management within the normotensive range, in addition to the diagnosis of hypertensive disorder of pregnancy based on a single BP measurement.

BP variability during pregnancy, a more sensitive and specific approach, can improve the identification and treatment of women at risk of future disease [26] and help in identifying pregnancies at high risk of poor birth outcomes [13–15]. Based on a large cluster of randomized trials in three less-developed countries, Magee et al. showed that high BP visit-to-visit variability measured by SD and ARV was associated with increased odds of the maternal and perinatal composites including maternal mortality and stillbirth [13]. Another large retrospective cohort study observed that DBP variability was associated with fetal distress, SGA, and low 1-min Apgar score [15]. In addition, Liu et al. found that DBP variability has shown superior predictive performance compared to SBP variability in predicting of SGA [14]. In our study, DBP variability but not SBP variability was associated with lower BSID scores and delayed psychomotor neurodevelopment. The coefficient of variation analysis revealed greater fluctuations in DBP compared to SBP throughout during pregnancy. This discrepancy may be attributed to the dynamic of peripheral blood vessels, particularly arterioles, which are abundant in the placenta. As such, DBP may serve as a more sensitive indicator of fetal blood perfusion and oxygenation. The observed association between DBP variability and PDI and MDI scores underscores the potential significance of vascular dynamics in fetal neurodevelopmental outcomes. These findings suggest that BP variability, especially for DBP, can be calculated and incorporated into the personalized maternal care process to provide high-definition medical care to high-risk pregnant women.

Previous studies have reported the association between hypertensive disorder of pregnancy and neurodevelopmental outcomes in offspring, possibly due to reduced fetal oxygenation resulting in structural brain alterations or inflammatory conditions [9]. The mechanism underlying associations between maternal BP patterns and child neurodevelopment is largely unknown, but it may involve to placental perfusion. Excessive BP variation can lead to placental hypoperfusion, triggering an increased production of pro-inflammatory cytokines and disrupting placental signaling and fetal circulation. This can alter the fetal neurodevelopmental trajectory and increase the risk of suboptimal neurodevelopmental outcomes [10, 27]. Adverse BP trajectories and high variability can also increase the odds of preterm neonates, who had a higher risk of neurodevelopmental delay. Even after adjusting for gestational weeks, the association between BP patterns and variability with neurodevelopmental delay remained significant, indicating an independent effect of BP patterns on early childhood neurodevelopment (Additional file 1: Table S1–S4). Further studies are needed to elucidate the exact pathways involved.

To our knowledge, this is the first study to examine the effects of patterns of BP changes during pregnancy on early childhood neurodevelopment. Our findings provide a scientific basis for rational guidance of healthcare during pregnancy. However, this study also has several limitations. First, all the participants were derived from a single source, limiting the generalization of findings to other populations. Second, although our model had adjusted for several demographic and clinical variables, there were some residual or unmeasured confounding factors related to the neurodevelopment that could bias our results. Further research is needed to confirm our findings and to explore potential underlying mechanisms.

## Conclusion

Our findings suggest that BP change patterns assessed by multi-trajectory and visit-to-visit variability were associated with lower BSID scores and delayed neurodevelopment. Health professionals should be aware of the influence of BP level and its oscillations during pregnancy on the risk of delayed neurodevelopment.

### Supplementary Information

Additional File 1: Table S1. Associations between multivariate trajectory groups for blood pressure and BSID scores. Table S2. Associations between multivariate trajectory groups for blood pressure and child neurodevelopment. Table S3. Associations between blood pressure variability and BSID scores. Table S4. Associations between blood pressure variability and child neurodevelopment

## Data Availability

The datasets used and/or analyzed during the current study are available from the corresponding author on reasonable request.

## Abbreviations

ARV: Average real variability
BMI: Body mass index
BSID: Bayley Scale of Infant Development
CV: Coefficient of variation
DBP: Diastolic blood pressure
GA: Gestational age
GBMTM: Group-based multi-trajectory modeling
GDM: Gestational diabetes mellitus
HDP: Hypertensive disorders of pregnancy
MDI: Mental development index
PDI: Psychomotor development index
SBP: Systolic blood pressure
SD: Standard deviation
SGA: Small for gestational age
