# Supplementary material for: Associations of prenatal blood pressure trajectory and variability with child neurodevelopment at 2 years old
Source: BMC Med. 2024 May 30;22:220. doi: 10.1186/s12916-024-03439-3 (PMC11140879; doi:10.1186/s12916-024-03439-3)
Supplement: Supplementary file 1 — Additional File 1: Table S1. Associations between multivariate trajectory groups for blood pressure and BSID scores. Table S2. Associations between multivariate trajectory groups for blood pressure and child neurodevelopment. Table S3. Associations between blood pressure variability and BSID scores. Table S4. Associations between blood pressure variability and child neurodevelopment [file 12916_2024_3439_MOESM1_ESM.docx]

| **Table S1** Associations between multivariate trajectory groups for blood pressure and BSID scores. | | | | |
| --- | --- | --- | --- | --- |
|  | PDI | | MDI | |
|  | Model 1 | Model 2 | Model 1 | Model 2 |
| Low-stable | ref | ref | ref | ref |
| Low-increasing | **-2.94 (-5.09, -0.80)** | **-2.93 (-5.07, -0.78)** | -0.81 (-3.35, 1.74) | -0.79 (-3.34, 1.76) |
| Moderate-increasing | **-3.24 (-5.34, -1.13)** | **-3.22 (-5.33, -1.11)** | -0.53 (-3.03, 1.97) | -0.52 (-3.02, 1.99) |
| Moderate-decreasing | **-3.12 (-5.21, -1.03)** | **-3.11 (-5.20, -1.02)** | -2.32 (-4.80, 0.16) | -2.31 (-4.79, 0.17) |
| High-stable | **-3.42 (-6.46, -0.39)** | **-3.40 (-6.43, -0.36)** | -2.60 (-6.20, 1.00) | -2.58 (-6.18, 1.02) |
| Model 1 was adjusted for maternal age, educational level, pre-pregnancy BMI, passive smoking status, infant sex, the number of BP measurements and gestational diabetes mellitus. Model 2 was additionally adjusted for gestational week. BSID, Bayley Scale of Infant Development; PDI, psychomotor development index; MDI. mental development index | | | | |

| **Table S2** Associations between multivariate trajectory groups for blood pressure and child neurodevelopment. | | | | |
| --- | --- | --- | --- | --- |
|  | Delayed psychomotor neurodevelopment | | Delayed mental neurodevelopment | |
|  | Model 1 | Model 2 | Model 1 | Model 2 |
| Low-stable | ref | ref | ref | ref |
| Low-increasing | **1.43 (1.01, 2.03)** | **1.43 (1.01, 2.04)** | 1.12 (0.79, 1.57) | 1.12 (0.80, 1.57) |
| Moderate-increasing | **1.48 (1.05, 2.08)** | **1.48 (1.05, 2.09)** | 1.11 (0.79, 1.55) | 1.11 (0.79, 1.56) |
| Moderate-decreasing | **1.45 (1.03, 2.04)** | **1.45 (1.03, 2.05)** | 1.22 (0.88, 1.69) | 1.22 (0.88, 1.69) |
| High-stable | 1.42 (0.87, 2.31) | 1.42 (0.87, 2.32) | 1.19 (0.75, 1.90) | 1.20 (0.75, 1.91) |
| Model 1 was adjusted for maternal age, educational level, pre-pregnancy BMI, passive smoking status, infant sex, the number of BP measurements and gestational diabetes mellitus. Model 2 was additionally adjusted for gestational week. | | | | |

| **Table S3** Associations between blood pressure variability and BSID scores. | | | | | |
| --- | --- | --- | --- | --- | --- |
|  | PDI | | MDI | | |
|  | Model 1 | Model 2 | Model 1 | | Model 2 |
| SBP-SD (mmHg) |  |  |  | |  |
| Low (<5.94) | ref | ref | ref | ref | |
| Intermediate (5.94-8.29) | 0.10 (-1.77, 1.96) | 0.10 (-1.77, 1.96) | 0.25 (-1.94, 2.44) | 0.25 (-1.94, 2.44) | |
| High (>8.29) | 0.13 (-1.71, 1.96) | 0.11 (-1.72, 1.94) | 0.01 (-2.14, 2.16) | 0.01 (-2.14, 2.15) | |
| SBP-CV |  |  |  |  | |
| Low (<5%) | ref | ref | ref | ref | |
| Intermediate (5%-7%) | 0.40 (-1.45, 2.26) | 0.40 (-1.46, 2.26) | -0.50 (-2.68, 1.68) | -0.50 (-2.68, 1.67) | |
| High (>7%) | 0.79 (-1.04, 2.62) | 0.78 (-1.06, 2.61) | 0.12 (-2.03, 2.27) | 0.12 (-2.04, 2.27) | |
| SBP-ARV (mmHg) |  |  |  |  | |
| Low (<7.0) | ref | ref | ref | ref | |
| Intermediate (7.0-10.4) | 0.96 (-0.89, 2.82) | 0.96 (-0.90, 2.81) | -0.74 (-2.92, 1.44) | -0.74 (-2.92, 1.44) | |
| High (>10.4) | -1.42 (-3.25, 0.42) | -1.42 (-3.25, 0.41) | -0.95 (-3.10, 1.20) | -0.95 (-3.11, 1.20) | |
| DBP-SD (mmHg) |  |  |  |  | |
| Low (<5.17) | ref | ref | ref | ref | |
| Intermediate (5.17-7.24) | -0.71 (-2.60, 1.18) | -0.72 (-2.61, 1.17) | -1.37 (-3.59, 0.85) | -1.37 (-3.59, 0.85) | |
| High (>7.24) | **-2.25 (-4.10, -0.40)** | **-2.27 (-4.12, -0.42)** | **-2.42 (-4.58, -0.25)** | **-2.42 (-4.59, -0.25)** | |
| DBP-CV |  |  |  |  | |
| Low (<7%) | ref | ref | ref | ref | |
| Intermediate (7%-11%) | -0.93 (-2.82, 0.96) | -0.93 (-2.82, 0.96) | -1.54 (-3.76, 0.68) | -1.54 (-3.76, 0.68) | |
| High (>11%) | **-2.44 (-4.30, -0.58)** | **-2.46 (-4.32, -0.60)** | **-2.28 (-4.46, -0.10)** | **-2.28 (-4.46, -0.10)** | |
| DBP-ARV (mmHg) |  |  |  |  | |
| Low (<6.0) | ref | ref | ref | ref | |
| Intermediate (6.0-9.0) | 0.94 (-0.94, 2.82) | 0.93 (-0.95, 2.81) | -0.92 (-3.13, 1.29) | -0.92 (-3.13, 1.29) | |
| High (>9.0) | -0.17 (-2.04, 1.70) | -0.19 (-2.06, 1.68) | -1.72 (-3.92, 0.47) | -1.73 (-3.92, 0.47) | |
| Each parameter was categorized into tertiles, with tertile 1, tertile 2, and tertile 3 considered as low, intermediate and high BP variability, respectively.  Model 1 was adjusted for maternal age, educational level, pre-pregnancy BMI, passive smoking status, infant sex, the number of BP measurements gestational diabetes mellitus and mean SBP or DBP. Model 2 was additionally adjusted for gestational week. BSID, Bayley Scale of Infant Development; PDI, psychomotor development index; MDI. mental development index; SBP, systolic blood pressure; DBP, diastolic blood pressure; SD, standard deviation; CV, coefficient of variation; ARV, average real variability. | | | | | |

| **Table S4** Associations between blood pressure variability and child neurodevelopment. | | | | |
| --- | --- | --- | --- | --- |
|  | Delayed psychomotor neurodevelopment | | Delayed mental neurodevelopment | |
|  | Model 1 | Model 2 | Model 1 | Model 2 |
| SBP-SD (mmHg) |  |  |  |  |
| Low (<5.94) | ref | ref | ref | ref |
| Intermediate (5.94-8.29) | 0.95 (0.72, 1.25) | 0.95 (0.72, 1.25) | 1.01 (0.76, 1.34) | 1.01 (0.76, 1.34) |
| High (>8.29) | 0.88 (0.67, 1.16) | 0.88 (0.67, 1.16) | 1.00 (0.75, 1.32) | 0.99 (0.75, 1.31) |
| SBP-CV |  |  |  |  |
| Low (<5%) | ref | ref | ref | ref |
| Intermediate (5%-7%) | 0.92 (0.70, 1.21) | 0.92 (0.70, 1.21) | 1.09 (0.82, 1.44) | 1.08 (0.82, 1.43) |
| High (>7%) | 0.85 (0.65, 1.12) | 0.85 (0.65, 1.12) | 0.94 (0.71, 1.25) | 0.94 (0.71, 1.24) |
| SBP-ARV (mmHg) |  |  |  |  |
| Low (<7.0) | ref | ref | ref | ref |
| Intermediate (7.0-10.4) | 0.98 (0.74, 1.30) | 0.98 (0.74, 1.30) | 1.14 (0.85, 1.51) | 1.13 (0.85, 1.51) |
| High (>10.4) | 1.05 (0.80, 1.38) | 1.05 (0.80, 1.38) | 1.09 (0.82, 1.45) | 1.09 (0.82, 1.45) |
| DBP-SD (mmHg) |  |  |  |  |
| Low (<5.17) | ref | ref | ref | ref |
| Intermediate (5.17-7.24) | 1.13 (0.84, 1.52) | 1.13 (0.84, 1.52) | 1.06 (0.79, 1.42) | 1.05 (0.79, 1.41) |
| High (>7.24) | **1.46 (1.11, 1.93)** | **1.46 (1.11, 1.93)** | 1.18 (0.89, 1.57) | 1.18 (0.89, 1.56) |
| DBP-CV |  |  |  |  |
| Low (<7%) | ref | ref | ref | ref |
| Intermediate (7%-11%) | 1.06 (0.79, 1.43) | 1.06 (0.79, 1.43) | 1.04 (0.78, 1.39) | 1.04 (0.78, 1.39) |
| High (>11%) | **1.54 (1.17, 2.03)** | **1.54 (1.17, 2.03)** | 1.17 (0.88, 1.55) | 1.16 (0.88, 1.54) |
| DBP-ARV (mmHg) |  |  |  |  |
| Low (<6.0) | ref | ref | ref | ref |
| Intermediate (6.0-9.0) | 0.82 (0.61, 1.08) | 0.82 (0.61, 1.08) | 1.10 (0.83, 1.47) | 1.11 (0.83, 1.48) |
| High (>9.0) | 1.03 (0.78, 1.35) | 1.03 (0.78, 1.35) | 1.09 (0.82, 1.44) | 1.08 (0.81, 1.44) |
| Each parameter was categorized into tertiles, with tertile 1, tertile 2, and tertile 3 considered as low, intermediate and high BP variability, respectively.  Model 1 was adjusted for maternal age, educational level, pre-pregnancy BMI, passive smoking status, infant sex, the number of BP measurements gestational diabetes mellitus and mean SBP or DBP. Model 2 was additionally adjusted for gestational week. SBP, systolic blood pressure; DBP, diastolic blood pressure; SD, standard deviation; CV, coefficient of variation; ARV, average real variability. | | | | |
